# *catena*-Poly[[dichloridozinc(II)]-μ-1,4-bis­(pyridin-2-ylmeth­oxy)benzene-κ^2^ *N*:*N*′]

**DOI:** 10.1107/S1600536811035963

**Published:** 2011-09-14

**Authors:** Ying Liu, Hong-Sen Zhang, Ming-Xing Hu, Guang-Feng Hou, Jin-Sheng Gao

**Affiliations:** a Department of Materials and Chemistry Engineering, Heilongjiang Institute of Technology, Harbin 150050, People’s Republic of China; b Modern Analysis, Test and Research Center, Heilongjiang Institute of Science and Technology, Harbin 150027, People’s Republic of China; c College of Chemistry and Materials Science, Heilongjiang University, Harbin 150080, People’s Republic of China

## Abstract

In the title compound, [ZnCl_2_(C_18_H_16_N_2_O_2_)]_*n*_, the Zn^II^ ion is tetra­hedrally coordinated by two Cl atoms and by two N atoms from different 1,4-bis­(pyridin-2-ylmeth­oxy)benzene ligands. The ligand shows a non-planar configuration, in which the dihedral angles between the two terminal pyridine rings and the linking benzene ring are 7.86 (12) and 70.74 (11)°. The flexible ligand coordinates to the Zn^II^ ions, generating an infinite chain propagating along [001].

## Related literature

For the synthesis and general background to flexible pyridyl-based ligands, see: Wang *et al.* (2007 ▶); Liu *et al.* (2010*a* ▶,*b* ▶)
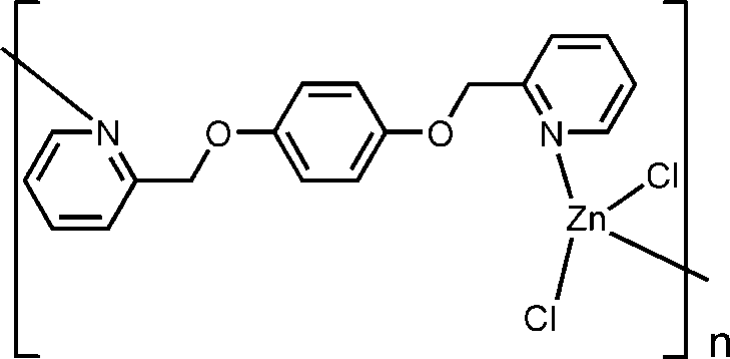


## Experimental

### 

#### Crystal data

[ZnCl_2_(C_18_H_16_N_2_O_2_)]
*M* *_r_* = 428.62
Triclinic, 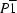

*a* = 8.8797 (18) Å
*b* = 10.458 (2) Å
*c* = 10.561 (2) Å
α = 87.55 (3)°
β = 73.50 (3)°
γ = 72.31 (3)°
*V* = 894.9 (3) Å^3^
*Z* = 2
Mo *K*α radiation
μ = 1.69 mm^−1^
*T* = 293 K
0.21 × 0.19 × 0.17 mm

#### Data collection

Rigaku R-AXIS RAPID diffractometer
Absorption correction: multi-scan (*ABSCOR*; Higashi, 1995 ▶) *T*_min_ = 0.719, *T*_max_ = 0.760
8755 measured reflections
4028 independent reflections
3222 reflections with *I* > 2σ(*I*)
*R*_int_ = 0.028

#### Refinement

*R*[*F* ^2^ > 2σ(*F* ^2^)] = 0.032
*wR*(*F* ^2^) = 0.073
*S* = 1.05
4028 reflections
226 parameters
H-atom parameters constrained
Δρ_max_ = 0.32 e Å^−3^
Δρ_min_ = −0.29 e Å^−3^

### 

Data collection: *RAPID-AUTO* (Rigaku, 1998 ▶); cell refinement: *RAPID-AUTO*; data reduction: *CrystalClear* (Rigaku/MSC, 2002 ▶); program(s) used to solve structure: *SHELXS97* (Sheldrick, 2008 ▶); program(s) used to refine structure: *SHELXL97* (Sheldrick, 2008 ▶); molecular graphics: *SHELXTL* (Sheldrick, 2008 ▶); software used to prepare material for publication: *SHELXL97*.

## Supplementary Material

Crystal structure: contains datablock(s) I, global. DOI: 10.1107/S1600536811035963/fj2447sup1.cif

Structure factors: contains datablock(s) I. DOI: 10.1107/S1600536811035963/fj2447Isup2.hkl

Additional supplementary materials:  crystallographic information; 3D view; checkCIF report

## Figures and Tables

**Fig. 1.**
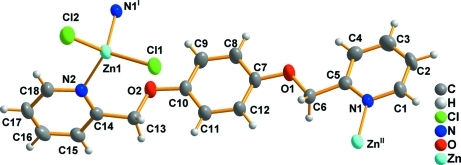
The molecular structure of the title compound, showing displacement ellipsoids at the 50% probability level for non-H atoms [Symmetry code I = x, y, z-1; II = x, y, z+1].

**Fig. 2.**
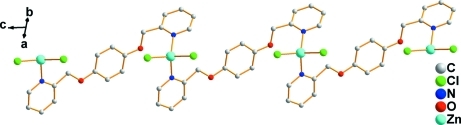
A partial packing view, showing the chain structure along b axis.

## supplementary crystallographic information

### Comment

The metal-organic frameworks are determined by many factors, in which the organic ligands as building blocks and the kinds of metal ions are most important. Many pyridyl-containing ligands with strong coordination ability and functional characteristics have been studied over the recent years (Wang *et al.*, 2007). The flexible bipyridyl ligands could react with transitional metals to construct the helical-like structures. Recently, as a continuration of previous works (Liu *et al.*, 2010*a*, *b*), we report the crystal structure of the title compound here.

In the title compound, [(ZnCl_2_)(C_18_H_16_N_2_O_2_)]_n_, each Zn^II^ atom is four-coordinated by two Cl atoms and two N atoms from different 1,4-bis(pyridin-2-ylmethoxy)benzene ligands to form a distorted tetrahedral geometry configuration. The Zn-Cl bond lengths are 2.228 (1) and 2.253 (1) Å, and Zn-N bond lengths are 2.090 (2) and 2.080 (2) Å. The Cl(1)-Zn-Cl(2) bond angle (110.45 °) and the N(1)-Zn-N(2) bond angle (111.03 °) are nearly equal to 120 ° in order to minimize the steric hindrance (Fig. 1, Table 1).

The ligand is oriented in a divergent fashion, in which the dihedral angles between two terminal pyridine rings with the linking benzene ring are 7.86 (12) and 70.74 (11)°. In the crystal packing, the flexible ligands link the Zn^II^ atoms to generate an infinite chain running along [001].

### Experimental

The 1,4-bis(pyridin-2-ylmethoxy)benzene ligand was synthesized as the reference method (Liu *et al.*, 2010*a*,*b*). The title compound was produced by reaction of ZnCl_2_ (0.50 mmol,0.068 g) in water (5 mL) and 1,4-bis(pyridin-2-ylmethoxy)benzene (0.5 mmol, 0.146 g) in 5 mL methanol under constant stirring, and filtered after stirring for about one hour. The filtate was maintained for about one week under the room temperature to give colorless block-like crystals suitable for X-ray analysis.

### Refinement

The anormal reflection datas (4 -5 6), (-3 2 0) and (2 -3 1) have been omitted during the refinement. H atoms bound to C atoms were placed in calculated positions and treated as riding on their parent atoms, with C—H = 0.93 Å (aromatic); C—H = 0.97 Å (methylene), and with *U*_iso_(H) = 1.2*U*_eq_(C).

### Crystal data

**Table:**

[ZnCl_2_(C_18_H_16_N_2_O_2_)]	*Z* = 2
*M**_r_* = 428.62	*F*(000) = 436
Triclinic, *P*1	*D*_x_ = 1.591 Mg m^−^^3^
Hall symbol: -P 1	Mo *K*α radiation, λ = 0.71073 Å
*a* = 8.8797 (18) Å	Cell parameters from 6941 reflections
*b* = 10.458 (2) Å	θ = 3.6–27.5°
*c* = 10.561 (2) Å	µ = 1.69 mm^−^^1^
α = 87.55 (3)°	*T* = 293 K
β = 73.50 (3)°	Block, colorless
γ = 72.31 (3)°	0.21 × 0.19 × 0.17 mm
*V* = 894.9 (3) Å^3^	

### Data collection

**Table:**

Rigaku R-AXIS RAPID diffractometer	4028 independent reflections
Radiation source: fine-focus sealed tube	3222 reflections with *I* > 2σ(*I*)
graphite	*R*_int_ = 0.028
ω scan	θ_max_ = 27.5°, θ_min_ = 3.6°
Absorption correction: multi-scan (*ABSCOR*; Higashi, 1995)	*h* = −10→11
*T*_min_ = 0.719, *T*_max_ = 0.760	*k* = −13→13
8755 measured reflections	*l* = −13→13

### Refinement

**Table:**

Refinement on *F*^2^	Primary atom site location: structure-invariant direct methods
Least-squares matrix: full	Secondary atom site location: difference Fourier map
*R*[*F*^2^ > 2σ(*F*^2^)] = 0.032	Hydrogen site location: inferred from neighbouring sites
*wR*(*F*^2^) = 0.073	H-atom parameters constrained
*S* = 1.05	*w* = 1/[σ^2^(*F*_o_^2^) + (0.0311*P*)^2^ + 0.1894*P*] where *P* = (*F*_o_^2^ + 2*F*_c_^2^)/3
4028 reflections	(Δ/σ)_max_ = 0.001
226 parameters	Δρ_max_ = 0.32 e Å^−^^3^
0 restraints	Δρ_min_ = −0.29 e Å^−^^3^

### Special details

**Table:**

Geometry. All esds (except the esd in the dihedral angle between two l.s. planes) are estimated using the full covariance matrix. The cell esds are taken into account individually in the estimation of esds in distances, angles and torsion angles; correlations between esds in cell parameters are only used when they are defined by crystal symmetry. An approximate (isotropic) treatment of cell esds is used for estimating esds involving l.s. planes.
Refinement. Refinement of F^2^ against ALL reflections. The weighted R-factor wR and goodness of fit S are based on F^2^, conventional R-factors R are based on F, with F set to zero for negative F^2^. The threshold expression of F^2^ > 2sigma(F^2^) is used only for calculating R-factors(gt) etc. and is not relevant to the choice of reflections for refinement. R-factors based on F^2^ are statistically about twice as large as those based on F, and R- factors based on ALL data will be even larger.

### Fractional atomic coordinates and isotropic or equivalent isotropic displacement parameters (Å^2^)

**Table:**

	*x*	*y*	*z*	*U*_iso_*/*U*_eq_	
C1	0.8929 (3)	0.5157 (2)	1.3377 (2)	0.0426 (5)	
H1	0.8933	0.5634	1.4099	0.051*	
C2	0.9801 (3)	0.3817 (2)	1.3198 (3)	0.0503 (6)	
H2	1.0380	0.3394	1.3786	0.060*	
C3	0.9800 (3)	0.3111 (2)	1.2128 (3)	0.0508 (6)	
H3	1.0393	0.2203	1.1976	0.061*	
C4	0.8915 (3)	0.3759 (2)	1.1287 (2)	0.0438 (6)	
H4	0.8894	0.3293	1.0565	0.053*	
C5	0.8052 (3)	0.5115 (2)	1.1526 (2)	0.0331 (5)	
C6	0.7058 (3)	0.5875 (2)	1.0652 (2)	0.0369 (5)	
H6A	0.5895	0.6153	1.1133	0.044*	
H6B	0.7376	0.6674	1.0367	0.044*	
C7	0.6566 (3)	0.5573 (2)	0.8599 (2)	0.0377 (5)	
C8	0.6904 (3)	0.4718 (2)	0.7515 (2)	0.0415 (5)	
H8	0.7590	0.3840	0.7478	0.050*	
C9	0.6227 (3)	0.5165 (2)	0.6498 (2)	0.0401 (5)	
H9	0.6442	0.4583	0.5782	0.048*	
C10	0.5228 (3)	0.6472 (2)	0.6532 (2)	0.0353 (5)	
C11	0.4855 (3)	0.7321 (2)	0.7619 (2)	0.0438 (6)	
H11	0.4157	0.8194	0.7658	0.053*	
C12	0.5525 (3)	0.6863 (2)	0.8651 (2)	0.0448 (6)	
H12	0.5270	0.7432	0.9386	0.054*	
C13	0.3676 (3)	0.8196 (2)	0.5444 (2)	0.0373 (5)	
H13A	0.4243	0.8803	0.5609	0.045*	
H13B	0.2650	0.8351	0.6145	0.045*	
C14	0.3322 (3)	0.8458 (2)	0.4139 (2)	0.0325 (5)	
C15	0.1734 (3)	0.8713 (2)	0.4044 (2)	0.0396 (5)	
H15	0.0900	0.8629	0.4778	0.047*	
C16	0.1382 (3)	0.9091 (2)	0.2865 (3)	0.0450 (6)	
H16	0.0322	0.9250	0.2792	0.054*	
C17	0.2624 (3)	0.9227 (2)	0.1811 (3)	0.0460 (6)	
H17	0.2419	0.9512	0.1013	0.055*	
C18	0.4196 (3)	0.8933 (2)	0.1949 (2)	0.0407 (5)	
H18	0.5040	0.9021	0.1225	0.049*	
Cl1	0.76914 (7)	0.83611 (6)	0.47450 (6)	0.04153 (14)	
Cl2	0.81884 (8)	0.89409 (6)	0.12085 (6)	0.04904 (16)	
N1	0.8066 (2)	0.58173 (17)	1.25574 (17)	0.0322 (4)	
N2	0.4562 (2)	0.85272 (17)	0.30782 (18)	0.0329 (4)	
O1	0.7344 (2)	0.50350 (16)	0.95442 (16)	0.0488 (4)	
O2	0.4678 (2)	0.68394 (15)	0.54293 (15)	0.0416 (4)	
Zn1	0.70586 (3)	0.78909 (2)	0.29601 (3)	0.03361 (9)	

### Atomic displacement parameters (Å^2^)

**Table:**

	*U*^11^	*U*^22^	*U*^33^	*U*^12^	*U*^13^	*U*^23^
C1	0.0489 (14)	0.0418 (12)	0.0389 (13)	−0.0072 (11)	−0.0224 (11)	0.0025 (10)
C2	0.0581 (16)	0.0458 (14)	0.0492 (16)	−0.0070 (12)	−0.0297 (13)	0.0140 (12)
C3	0.0563 (16)	0.0348 (12)	0.0540 (17)	−0.0014 (12)	−0.0184 (13)	0.0058 (11)
C4	0.0527 (14)	0.0372 (12)	0.0387 (14)	−0.0083 (11)	−0.0142 (11)	−0.0014 (10)
C5	0.0334 (11)	0.0354 (11)	0.0295 (11)	−0.0095 (9)	−0.0089 (9)	0.0034 (9)
C6	0.0449 (13)	0.0384 (11)	0.0287 (12)	−0.0084 (10)	−0.0169 (10)	−0.0003 (9)
C7	0.0503 (13)	0.0351 (11)	0.0323 (12)	−0.0120 (11)	−0.0201 (11)	0.0018 (9)
C8	0.0590 (15)	0.0292 (11)	0.0384 (13)	−0.0087 (11)	−0.0219 (12)	0.0011 (9)
C9	0.0594 (15)	0.0318 (11)	0.0343 (13)	−0.0152 (11)	−0.0192 (11)	−0.0026 (9)
C10	0.0442 (13)	0.0363 (11)	0.0307 (12)	−0.0130 (10)	−0.0181 (10)	0.0022 (9)
C11	0.0543 (15)	0.0378 (12)	0.0378 (13)	−0.0029 (11)	−0.0218 (12)	−0.0034 (10)
C12	0.0567 (15)	0.0397 (12)	0.0347 (13)	0.0002 (11)	−0.0226 (12)	−0.0082 (10)
C13	0.0384 (12)	0.0391 (11)	0.0310 (12)	−0.0038 (10)	−0.0127 (10)	−0.0028 (9)
C14	0.0351 (11)	0.0269 (10)	0.0356 (12)	−0.0034 (9)	−0.0161 (10)	−0.0033 (8)
C15	0.0367 (12)	0.0382 (12)	0.0448 (14)	−0.0080 (10)	−0.0164 (10)	−0.0017 (10)
C16	0.0382 (13)	0.0423 (13)	0.0575 (16)	−0.0031 (11)	−0.0271 (12)	−0.0031 (11)
C17	0.0532 (15)	0.0444 (13)	0.0433 (14)	−0.0020 (12)	−0.0321 (13)	0.0031 (11)
C18	0.0440 (13)	0.0407 (12)	0.0342 (13)	−0.0026 (11)	−0.0175 (11)	0.0029 (10)
Cl1	0.0482 (3)	0.0434 (3)	0.0426 (3)	−0.0150 (3)	−0.0264 (3)	0.0013 (2)
Cl2	0.0539 (4)	0.0542 (3)	0.0416 (3)	−0.0198 (3)	−0.0154 (3)	0.0129 (3)
N1	0.0331 (9)	0.0344 (9)	0.0291 (9)	−0.0069 (8)	−0.0127 (8)	0.0033 (7)
N2	0.0343 (9)	0.0324 (9)	0.0307 (10)	−0.0022 (8)	−0.0155 (8)	−0.0011 (7)
O1	0.0704 (12)	0.0377 (8)	0.0383 (10)	−0.0005 (8)	−0.0320 (9)	−0.0043 (7)
O2	0.0607 (10)	0.0338 (8)	0.0359 (9)	−0.0084 (8)	−0.0287 (8)	−0.0001 (6)
Zn1	0.03523 (15)	0.03499 (14)	0.03301 (15)	−0.00713 (11)	−0.01731 (11)	0.00198 (10)

### Geometric parameters (Å, °)

**Table:**

C1—N1	1.348 (3)	C11—C12	1.387 (3)
C1—C2	1.371 (3)	C11—H11	0.9300
C1—H1	0.9300	C12—H12	0.9300
C2—C3	1.376 (4)	C13—O2	1.426 (3)
C2—H2	0.9300	C13—C14	1.495 (3)
C3—C4	1.374 (3)	C13—H13A	0.9700
C3—H3	0.9300	C13—H13B	0.9700
C4—C5	1.387 (3)	C14—N2	1.346 (3)
C4—H4	0.9300	C14—C15	1.385 (3)
C5—N1	1.344 (3)	C15—C16	1.381 (3)
C5—C6	1.496 (3)	C15—H15	0.9300
C6—O1	1.410 (3)	C16—C17	1.364 (4)
C6—H6A	0.9700	C16—H16	0.9300
C6—H6B	0.9700	C17—C18	1.384 (3)
C7—C12	1.377 (3)	C17—H17	0.9300
C7—O1	1.378 (3)	C18—N2	1.341 (3)
C7—C8	1.390 (3)	C18—H18	0.9300
C8—C9	1.373 (3)	Cl1—Zn1	2.2271 (7)
C8—H8	0.9300	Cl2—Zn1	2.2530 (10)
C9—C10	1.380 (3)	N1—Zn1^i^	2.0898 (18)
C9—H9	0.9300	N2—Zn1	2.0803 (18)
C10—C11	1.381 (3)	Zn1—N1^ii^	2.0898 (18)
C10—O2	1.383 (3)		
			
N1—C1—C2	123.2 (2)	C7—C12—H12	119.7
N1—C1—H1	118.4	C11—C12—H12	119.7
C2—C1—H1	118.4	O2—C13—C14	109.20 (17)
C1—C2—C3	118.5 (2)	O2—C13—H13A	109.8
C1—C2—H2	120.8	C14—C13—H13A	109.8
C3—C2—H2	120.8	O2—C13—H13B	109.8
C4—C3—C2	119.4 (2)	C14—C13—H13B	109.8
C4—C3—H3	120.3	H13A—C13—H13B	108.3
C2—C3—H3	120.3	N2—C14—C15	120.9 (2)
C3—C4—C5	119.4 (2)	N2—C14—C13	118.31 (19)
C3—C4—H4	120.3	C15—C14—C13	120.6 (2)
C5—C4—H4	120.3	C16—C15—C14	120.4 (2)
N1—C5—C4	121.7 (2)	C16—C15—H15	119.8
N1—C5—C6	116.45 (18)	C14—C15—H15	119.8
C4—C5—C6	121.9 (2)	C17—C16—C15	118.5 (2)
O1—C6—C5	108.69 (18)	C17—C16—H16	120.8
O1—C6—H6A	110.0	C15—C16—H16	120.8
C5—C6—H6A	110.0	C16—C17—C18	118.8 (2)
O1—C6—H6B	110.0	C16—C17—H17	120.6
C5—C6—H6B	110.0	C18—C17—H17	120.6
H6A—C6—H6B	108.3	N2—C18—C17	123.1 (2)
C12—C7—O1	125.3 (2)	N2—C18—H18	118.4
C12—C7—C8	119.3 (2)	C17—C18—H18	118.4
O1—C7—C8	115.4 (2)	C5—N1—C1	117.94 (18)
C9—C8—C7	120.1 (2)	C5—N1—Zn1^i^	127.03 (14)
C9—C8—H8	119.9	C1—N1—Zn1^i^	114.79 (14)
C7—C8—H8	119.9	C18—N2—C14	118.08 (19)
C8—C9—C10	120.5 (2)	C18—N2—Zn1	115.68 (15)
C8—C9—H9	119.8	C14—N2—Zn1	125.98 (14)
C10—C9—H9	119.8	C7—O1—C6	117.33 (17)
C9—C10—C11	119.8 (2)	C10—O2—C13	116.16 (17)
C9—C10—O2	115.81 (19)	N2—Zn1—N1^ii^	111.05 (7)
C11—C10—O2	124.4 (2)	N2—Zn1—Cl1	115.74 (6)
C10—C11—C12	119.6 (2)	N1^ii^—Zn1—Cl1	107.18 (6)
C10—C11—H11	120.2	N2—Zn1—Cl2	103.67 (6)
C12—C11—H11	120.2	N1^ii^—Zn1—Cl2	108.57 (6)
C7—C12—C11	120.6 (2)	Cl1—Zn1—Cl2	110.47 (3)

Symmetry codes: (i) *x*, *y*, *z*+1; (ii) *x*, *y*, *z*−1.
